# HIV virological suppression influences response to the AS03-adjuvanted monovalent pandemic influenza A H1N1 vaccine in HIV-infected children

**DOI:** 10.1111/irv.12243

**Published:** 2014-02-18

**Authors:** Timothy R Leahy, Michelle Goode, Paul Lynam, Patrick J Gavin, Karina M Butler

**Affiliations:** a Department of Paediatric Infectious Diseases and Immunology, Our Lady's Children's HospitalDublin, Ireland; b Department of Haematology, Our Lady's Children's HospitalDublin, Ireland

**Keywords:** AS03 adjuvant, HIV, pandemic H1N1 influenza, vaccination

## Abstract

**Design:**

Children with HIV are especially susceptible to complications from influenza infection, and effective vaccines are central to reducing disease burden in this population. We undertook a prospective, observational study to investigate the safety and immunogenicity of the inactivated split-virion AS03-adjuvanted pandemic H1N1(2009) vaccine in children with HIV.

**Setting:**

National referral centre for Paediatric HIV in Ireland.

**Sample:**

Twenty four children with HIV were recruited consecutively and received two doses of the vaccine. The serological response was measured before each vaccine dose (Day 0 and Day 28) and 2 months after the booster dose. Antibody titres were measured using a haemagglutination inhibition (HAI) assay. Seroprotection was defined as a HAI titre ≥ 1:40; seroconversion was defined as a ≥ fourfold increase in antibody titre and a postvaccination titre ≥ 1:40.

**Main outcome measures:**

The seroconversion rates after prime and booster doses were 75% and 71%, respectively. HIV virological suppression at the time of immunization was associated with a significantly increased seroconversion rate (*P* = 0·009), magnitude of serological response (*P* = 0·02) and presence of seroprotective HAI titres (*P* = 0·017) two months after the booster dose. No other factor was significantly associated with the seroconversion/seroprotection rate. No serious adverse effects were reported. Vaccination had no impact on HIV disease progression. The AS03-adjuvanted pandemic H1N1 vaccine appears to be safe and immunogenic among HIV-infected children. A robust serological response appears to be optimized by adherence to a HAART regimen delivering virological suppression.

## Introduction

The influenza viruses are responsible for annual winter epidemics of respiratory tract infections, particularly in temperate regions. In addition, four influenza pandemics have been documented since 1900.^1^ The most recent pandemic occurred in 2009 and was caused by a novel H1N1 influenza A virus (pH1N1), first identified in Mexico.^2^ Influenza infection is associated with a greater infection rate, greater morbidity and higher mortality among those living with HIV.^3^–^5^ The reported impact of pH1N1 on people living with HIV/AIDS has been variable.^6^ A seroprevalence study conducted in Australia showed no difference in serological evidence of pH1N1 infection based on HIV status.^7^ Several observational studies have shown no difference in clinical course of pH1N1 infection between HIV-positive and HIV-negative patients, either adults or children.^8^–^11^ On the other hand, a study of hospitalizations due to pH1N1 conducted in the US reported a disproportionately high admission rate among HIV-positive patients, while an observational study conducted in Mexico city documented poorer outcome among HIV-positive patients not on highly active antiretroviral treatment (HAART) and with PCR-detectable HIV.^12^,^13^ The impact of pH1N1 infection therefore appears to be at least partially influenced by treatment and immunovirological status.

Vaccination represents an important strategy to reduce influenza disease burden. Studies on the immunogenicity of pH1N1 vaccination among HIV-positive adults have demonstrated better immunogenicity with adjuvanted vaccines, and a more sustained response after prime and booster vaccinations.^6^,^14^,^15^ Similar data have emerged from studies on both the unadjuvanted and the mf59-adjuvanted pH1N1 vaccine in HIV-infected children.^16^–^19^ In July 2009, shortly after the pandemic was declared, a Strategic Advisory Group of Experts on Vaccination strongly advocated the use of a vaccine against pH1N1 among vulnerable groups including people living with HIV.^20^ Large-scale vaccination against pH1N1 was undertaken in Ireland using an inactivated, split-virion AS03 (squalene oil emulsion) adjuvanted vaccine (Pandemrix). Okike *et al*.^21^ have previously examined the safety and immunogenicity of this vaccine in HIV-positive children, but did not compare the response after the prime and booster vaccine dose.

The aim of this study was to investigate the safety and immunogenicity of the AS03-adjuvanted pH1N1 vaccine among children with HIV, to compare the response after the prime and booster dose, to explore factors predicting serological response to the vaccine and the impact of vaccination on the immunovirological status of those vaccinated.

## Methods

### Study participants

The study was undertaken in Our Lady's Children's Hospital Crumlin (OLCHC), Dublin, Ireland, during the influenza season of 2009/10. This hospital is a tertiary referral paediatric hospital and serves as the national centre for the treatment of children with HIV. Following ethical approval by the institutional ethics committee, HIV-infected children above the age of 6 months were eligible for recruitment and were enrolled consecutively through the paediatric infectious diseases Rainbow Clinic.

Written informed consent was obtained from parents/guardians prior to enrolment. Exclusion criteria included documented pH1N1 infection or documented allergy to components of the vaccine (e.g. egg, gelatine, gentamicin). Participants with influenza-like illness (ILI) symptoms or signs/symptoms suggestive of respiratory tract infection during the course of the study had nasopharyngeal aspirates taken and screened for the presence of pH1N1 using both direct fluorescent antibody (DFA) staining and PCR.

### Vaccine description and immunization schedule

The vaccination schedule comprised two doses of the inactivated, split-virion, AS03-adjuvanted H1N1 vaccine (Pandemrix; GlaxoSmithKline, Brentford, Middlesex, UK). Pandemrix was derived from the A/California/7/2009(H1N1) v-like strain antigen (New York Medical College x-179A). The doses were administered at a 28-day interval. The vaccinations were given into the deltoid muscle. Children below the age of 13 years received 0·25 ml per dose, whereas older children received the adult dose of 0·5 ml in keeping with national guidelines issued by the Health Protection Surveillance centre. Participants were requested to report any adverse effects to the vaccination by telephone and were questioned on possible adverse effects at follow-up visits.

### Data and blood sample collection

Relevant demographic and medical data on each participant were collected by interview and examination of medical records. The CD4 count was performed using a Becton Dickinson FACSCalibur flow cytometer, while the HIV RNA PCR testing was performed at the National Virus Reference Laboratory, Belfield, University College Dublin, using a Roche COBAS assay with a lower limit of detection of 50 copies/ml. A blood sample for measurement of serological response was collected on the day of each vaccination (Day 0 and Day 28), and a final sample was obtained 2 months after the booster vaccine dose. Laboratory personnel were blinded to the participants' clinical details and vaccination status.

### Haemagglutination inhibition assay (HAI): laboratory methods

Haemagglutination inhibition titres were determined in GSK biological laboratories in Belgium. Each HAI titre represents the geometric mean titre (GMT) of two measurements performed in the same run on two separate microtitre plates. Results were reported as the inverse of the highest positive HAI dilution. Negative samples were, for statistical purposes, assigned a value of 1:5.

### Immunological endpoints

The primary immunological endpoint was the proportion of children who seroconverted to vaccination (i.e. HAI titre ≥ 1:40 and GMT-fold increase ≥4 post-vaccination). Secondary immunological endpoints included the seroprotection rate (i.e. proportion with HAI titre ≥ 1:40) and the mean fold increase (MFI) in GMT.

### Statistical analysis

The sample size was based on our single-centre recruitment capacity. Descriptive statistics were calculated for the study participants' baseline characteristics, outcome variables and other covariates of interest. The influence of age and baseline CD4 count on the likelihood of seroconversion was assessed using the Mann–Whitney nonparametric test. Univariate analysis of the seroconversion rate by patient characteristic was performed using Fisher's exact test/Pearson's chi-squared test as appropriate. The independence of any risk factors significantly associated with seroconversion was tested by multivariate analysis using a nominal logistic regression model. The Wilcoxon rank sum test was used to compare the pre-vaccination and MFI in HAI titres between patient subgroups. anova of repeated measures was conducted by group on any factor associated with a statistically significant dose-on-dose increase in response to vaccination. The impact of vaccination on HIV immunological status was assessed by comparing the mean CD4 count pre- and post-vaccination using the Friedman nonparametric test for repeated measures and Dunn's multiple comparison test. Ninety-five per cent confidence intervals and a *P*-value of 5% were employed for all statistical calculations. Statistical analysis was performed using JMP-4 software (SAS institute, Cary, NJ, USA).

## Results

Twenty-five HIV-infected children and adolescents were successfully enrolled in the study. One child developed proven pH1N1 infection between the first and second dose of the vaccine and was excluded from the analysis. The baseline characteristics of the remaining 24 children are summarized in Table 1. The median age on date of prime vaccination was 10·2 years (range 2·7–16·8 years). Only one child was <5 years old. The mean CD4 count at enrolment was 824 cells/µl (range 364–1416 cells/µl). Twenty children (83·3%) were on HAART, of whom 17 (85%) were virologically suppressed with undetectable HIV RNA.

**Table 1:** Baseline patient characteristics

Characteristic	Variable	*N* (%)
Age	>13 years	9 (37·5)
	<13 years	15 (62·5)
Gender	Male	13 (54·2)
	Female	11 (45·8)
Ethnicity	African	18 (75)
	Caucasian	5 (20·8)
	Mixed	1 (4·2)
CDC clinical stage	N/A	8 (33·3)
	B	9 (37·5)
	C	7 (29·2)
CDC immunological stage	1	2 (8·3)
	2	13 (54·2)
	3	9 (37·5)
On HAART	Yes	20 (83·3)
	PI-based	14 (58·3)
	NNRTI-based	6 (25)
	No	4 (16·7)
Virological suppression	Yes	17 (70·8)
	No	7 (29·2)

All 24 children received both doses of the vaccine, and all had HAI titres taken as per study protocol. The median interval from prime to booster dose of the vaccine was 23 days (range 20–29 days), and the median interval from booster dose to final HAI titre measurement was 68 days (range 41–98 days). Seven children (29%) had HAI titres ≥ 1:40 before vaccination. Six of these seven children boosted post-vaccination, and all seven maintained seroprotective titres.

The overall seroconversion and seroprotection rates were 75% and 88%, respectively, after the prime dose. The seroconversion and seroprotection rates were 71% and 88%, respectively, after the booster dose. Children who had sustained seroconversion (*n* = 17) after the booster dose of vaccine did not differ significantly from those who did not sustain seroconversion (*n* = 7) by age (11 versus 10·7 year, *P* = 0·73) or by baseline CD4 count (873 versus 712 cells/µl, *P* = 0·39).

**Figure 1:**
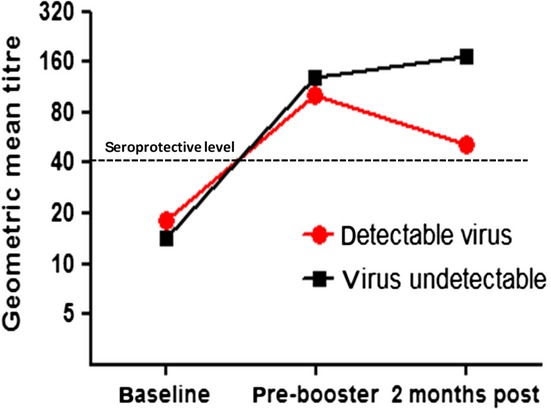
Serological response to vaccination by HIV virological status.

The impact of baseline patient characteristics on the seroconversion and seroprotection rates is summarized in Table 2. Seventeen of twenty children on HAART were fully virologically suppressed. Of these 17, 15 sustained seroconversion after two doses of the vaccine. Of the seven children who were either treatment naive or on HAART but not virologically suppressed, five seroconverted after one dose, but only two maintained seroconversion 2 months after the second dose. Virological suppression was statistically significantly associated with sustained seroconversion in the study cohort as a whole (*P* = 0·009) and approached statistical significance (*P* = 0·09) in the subset of children on HAART. All eight children with a WHO clinical stage of N/A at diagnosis seroconverted, whereas nine of sixteen with a clinical stage of B/C seroconverted, suggesting an association, albeit one that did not reach statistical significance (*P* = 0·054). However, this association was not upheld in multivariate analysis (Table 3) which confirmed that virological suppression alone was strongly associated with seroconversion (odds ratio of 18·7, *P-*value 0·03). HIV virological suppression was also the only factor significantly associated with the magnitude of the immunological response (*P* = 0·02) as measured by both absolute and mean fold increase (MFI) in HAI titres 2 months post-booster vaccination (Table 2 and Figure 1).

**Table 2:** Univariate analysis by patient characteristic of seroconversion and seroprotection rates and mean fold increase (MFI) in geometric mean titre after prime and booster vaccine dose

Characteristic	Variable	After prime vaccine dose						After booster vaccine dose					
	
		Seroconversion *n/N* (%)	*P*	Seroprotection *n/N* (%)	*P*	MFI titres mean(95% CI)	*P*	Seroconversion *n/N* (%)	*P*	Seroprotection *n/N* (%)	*P*	MFI titres mean (95% CI)	*P*
Total	–	18/24 (75)	–	21/24 (88)	–	8·0 (4·9–13·1)	–	17/24 (71)	–	21/24 (88)	–	8·0 (4·4–14·5)	–
Gender	Female	7/11 (64)	0·37	9/11 (82)	0·58	11·3 (5·7–22·6)	0·09	7/11 (64)	0·66	9/11 (82)	0·58	12·3 (5–29·9)	0·14
	Male	11/13 (85)		12/13 (92)		5·3 (2·5–11·3)		10/13 (77)		12/13 (92)		4·8 (2·2–10·8)	
Ethnicity	African	15/18 (83)	0·29	17/18 (94)	0·11	8·8 (4·8–16·1)	0·48	13/18 (72)	>0·99	16/18 (89)	0·54	6·9 (3·6–13·1)	0·33
	Caucasian	3/5 (60)		3/5 (60)		7·5 (2·0–27·5)		4/5 (80)		4/5 (80)		18·4 (2·4–143)	
CDC clinical stage	N/A	8/8 (100)	0·07	8/8 (100)	0·53	13·4 (7·4–24·5)	0·15	8/8 (100)	0·05	8/8 (100)	0·53	12·9 (5·7–29·2)	0·14
	B/C	10/16 (63)		13/16 (81)		6·2 (3·1–12·2)		9/16 (56)		13/16 (81)		6·3 (2·8–14·4)	
HAART	No	3/4 (75)	>0·99	4/4 (100)	>0·99	8·9 (5·3–14·9)	0·37	1/4 (25)	0·06	3/4 (75)	0·44	10·6 (6·0–18·6)	0·07
	Yes	15/20 (75)		17/20 (85)		4·8 (0·4–53)		16/20 (80)		18/20 (90)		2 (0·2–29·2)	
HAART regimen	NNRTI-based	5/6 (83)	0·83	6/6 (100)	>0·99	8·2 (4·2–16·1)	0·77	5/6 (83)	0·83	6/6 (100)	>0·99	10·5 (5–22·2)	0·77
	PI-based	10/14 (71)		11/14 (79)		10·7 (3·6–31·8)		11/14 (79)		12/14 (86)		10·7 (3·4–33·3)	
HIV virological suppression	Yes	13/17 (77)	>0·99	15/17 (88)	>0·99	9·2 (5·1–16·7)	0·34	15/17 (88)	0·009*	17/17 (100)	0·017*	12·3 (6·5–23·4)	0·02*
	No	5/7 (71)		6/7 (86)		5·7 (1·8–17·7)		2/7 (29)		4/7 (57)		2·8 (0·9–9·2)	
Vaccine dose	0·25 ml	12/15 (80)	0·64	14/15 (93)	0·53	7·1 (3·2–15·9)	0·42	10/15 (67)	0·67	13/15 (87)	>0·99	11·8 (3·6–38)	0·63
	0·5 ml	6/9 (67)		7/9 (78)		8·6 (4·2–17·4)		7/9 (78)		8/9 (89)		6·3 (3·0–13·3)	

*P*-values calculated using Fisher's exact test or the chi-squared test for seroconversion/seroprotection rates and Wilcoxon rank sum test for MFI in geometric mean titre.

* Statistical significance with a *P*-value <0·05.

**Table 3:** Multivariate analysis of seroconversion rate by baseline characteristics after two doses of vaccine

Characteristic	Seroconversion rate (%)	Odds ratio (95% CI)	*P*-value
CDC clinical stage			
N/A	8/8 (100)	13·4 (0·66–272)	0·96
B/C	9/16 (56)		
HIV virological suppression			
Yes	15/17 (88)	18·7 (2–170·3)	0·03*
No	2/7 (29)		

Odds ratio calculated using Fisher's exact test. *P*-value calculated using Wald chi-squared test.

* statistical significance.

The mean CD4 count had dropped 3 months post-vaccination to 742 cells/µl (range 311–1438 cells/µl) but recovered to 768 cells/µl (range 340–1631 cells/µl) 6 months post-date of first vaccination. The difference between mean CD4 counts on each of these occasions was not statistically significant (*P*-value = 0·42). The proportion of children with detectable HIV was 12/24 three months post-vaccination and 10/24 six months post-vaccination; the difference in proportions from pre-vaccination was not statistically significant (*P*-value = 0·33). The magnitude of these ‘viral blips’ in the five children previously virologically suppressed was small (median 152 copies/ml, range 52–794 copies). No alterations in treatment regimen were indicated during the course of the study as a result of these changes.

Adverse reactions to vaccination are documented in Table 4. Grade 2 injection site pain was reported in 76% and 67%, respectively, after prime and booster vaccinations. No other significant adverse reactions were reported.

**Table 4:** Adverse reactions to vaccination

Adverse effect	Severity score	Vaccine dose	

		Prime	Booster
Fever	Grade 2+ (>38·7°C)	0	0
	Grade 1 (37·7–38·6°C)	1	0
	No fever	23	24
	Not recorded	1	1
Injection site pain	Grade 2+ (interference with activities)	19	16
	Grade 1 (minimal limitation of use)	5	8
	None	1	1
Local reaction	Grade 1 (erythema/induration, ≤2·5 cm)	1	0
	None	24	24
	Not recorded	0	1
Analgesia required	Yes	8	7
	No	16	17

## Discussion

Children living with HIV, particularly those not on effective HAART are particularly susceptible to influenza infection and its complications; consequently, new influenza vaccines should be assessed for their immunogenicity in this vulnerable population. The Committee for Proprietary Medicinal Products (CPMP) guidelines issued in 1997 suggest a minimum immunogenicity standard of a seroconversion and seroprotection rate of 40% and 70%, respectively, by Day 21 post-vaccination among adult patients.^22^ The AS03-adjuvanted monovalent pH1N1 vaccine met and exceeded these targets in our study cohort. The seroconversion rates of 75% after the prime dose and 71% after the booster dose compared favourably with rates reported for the unadjuvanted vaccine in Thai children,^19^ and the mf59-adjuvanted monovalent pH1N1 vaccine in Italian children with HIV.^17^ Other studies on the immunogenicity of the mf59- and AS03-adjuvanted pH1N1 vaccine in children with HIV, however, reported higher seroconversion and seroprotection rates.^16^,^18^,^21^

Seven children (29%) had protective antibody titres to pH1N1 at baseline, reflecting a trend seen in other pH1N1 immunogenicity studies, and signifying either cross-reactivity with antibody to seasonal influenza or previous infection.^6^ In contrast to other studies in adults and children with HIV, the booster dose of vaccine did not appear to increase either the overall seroconversion rate or the magnitude of the immunological response in our study participants.^14^,^15^,^17^

A number of previous studies have correlated poor immunological status at vaccination with poor vaccine response.^15^,^23^ Although there was a trend towards a lower baseline CD4 count among those children who did not seroconvert in our study cohort, this did not reach statistical significance (*P* = 0·39). HIV virological suppression at vaccination was significantly associated with a greater likelihood of sustained seroconversion (*P* = 0·009) and seroprotective antibody levels (*P* = 0·017) measured 2 months after the booster dose and was associated with a significantly greater magnitude of immunological response as measured by fold increase in GMT (*P* = 0·02), an effect that was magnified with time (Figure 1). HIV virological suppression has previously been associated with a better immunological response to the pH1N1 vaccine in HIV-positive adults.^6^,^24^ These data suggest that a robust and sustained serological response to the pH1N1 vaccine in HIV-positive children depends on adherence to a HAART regimen that delivers both immunological reconstitution and virological suppression.

The mean absolute CD4 count measured 3 months post-vaccination dipped slightly; this difference was not statistically significant, did not equate with any clinical deterioration and did not necessitate any change in management. The mean CD4 count after 6 months had increased back towards the baseline. A similar transient dip in CD4 count was seen in a prospective observational study among 51 HIV-positive children in Rio De Janeiro after receiving seasonal influenza vaccine.^25^ Some of our study participants experienced viral ‘blips’ post-vaccination. While not of sufficient magnitude to merit alteration in management, it may suggest mobilization of a latently infected CD4+ memory cell reservoir.^26^ Overall, however, as has been previously confirmed in well-designed, prospective, longitudinal studies, influenza vaccination did not negatively impact on HIV disease progression.^27^–^29^

A significant proportion of our study participants experienced local pain and local reactions to the AS03-adjuvanted vaccine (Table 4); other studies have documented similar findings.^6^ Overall, the pH1N1 vaccines appear to have been more reactogenic than seasonal influenza vaccines.^6^ None of our study participants suffered any debilitating or longer-lasting adverse effects, and only one in three had injection site pain that merited administration of analgesia. The quality of data collected is robust, and we achieved 100% follow-up on our enrolled patients. The study is, however, severely limited by the small sample size, leading to broad confidence intervals, which, in turn, weaken the validity of our findings.

In conclusion, the AS03-adjuvanted pH1N1 vaccine appears to be both safe and immunogenic in children living with HIV. Children on HAART with full virological suppression were more likely to sustain a robust serological response to the vaccine, whereas children with detectable HIV RNA demonstrated earlier decay of serological response. These findings may have implications for future vaccination strategies, not only against pH1N1, but also against novel or emerging strains of influenza in this vulnerable population.
